# Improving the use of research evidence in guideline development: 9. Grading evidence and recommendations

**DOI:** 10.1186/1478-4505-4-21

**Published:** 2006-12-05

**Authors:** Holger J Schünemann, Atle Fretheim, Andrew D Oxman

**Affiliations:** 1 INFORMA/CLARITY Research Group, S.C. Epidemiologia, Istitituto Regina Elena, Via Elio Chianesi 53, 00144 Rome, Italy; 2 Norwegian Knowledge Centre for the Health Services, P.O. Box 7004, St. Olavs plass, N-0130 Oslo, Norway

## Abstract

**Background:**

The World Health Organization (WHO), like many other organisations around the world, has recognised the need to use more rigorous processes to ensure that health care recommendations are informed by the best available research evidence. This is the ninth of a series of 16 reviews that have been prepared as background for advice from the WHO Advisory Committee on Health Research to WHO on how to achieve this.

**Objectives:**

We reviewed the literature on grading evidence and recommendations in guidelines.

**Methods:**

We searched PubMed and three databases of methodological studies for existing systematic reviews and relevant methodological research. We did not conduct a full systematic review ourselves. Our conclusions are based on the available evidence, consideration of what WHO and other organisations are doing and logical arguments.

**Key questions and answers:**

Should WHO grade the quality of evidence and the strength of recommendations?

• Users of recommendations need to know how much confidence they can place in the underlying evidence and the recommendations. The degree of confidence depends on a number of factors and requires complex judgments. These judgments should be made explicitly in WHO recommendations. A systematic and explicit approach to making judgments about the quality of evidence and the strength of recommendations can help to prevent errors, facilitate critical appraisal of these judgments, and can help to improve communication of this information.

What criteria should be used to grade evidence and recommendations?

• Both the quality of evidence and the strength of recommendations should be graded. The criteria used to grade the strength of recommendations should include the quality of the underlying evidence, but should not be limited to that.

• The approach to grading should be one that has wide international support and is suitable for a wide range of different types of recommendations. The Grading of Recommendations Assessment, Development and Evaluation (GRADE) approach, which is currently suggested in the Guidelines for WHO Guidelines, is being used by an increasing number of other organizations internationally. It should be used more consistently by WHO. Further developments of this approach should ensure its wide applicability.

Should WHO use the same grading system for all of its recommendations?

• Although there are arguments for and against using the same grading system across a wide range of different types of recommendations, WHO should use a uniform grading system to prevent confusion for developers and users of recommendations.

## Background

The World Health Organization (WHO), like many other organisations around the world, has recognised the need to use more rigorous processes to ensure that health care recommendations are informed by the best available research evidence. This is the ninth of a series of 16 reviews that have been prepared as background for advice from the WHO Advisory Committee on Health Research to WHO on how to achieve this.

For over 25 years a growing number of organisations have employed various systems to grade the quality of evidence (sometimes called levels of evidence) and the strength of recommendations [1]. Unfortunately, different organisations use various grading systems, which may lead to confusion among consumers.

Groups making recommendations always make judgements about the quality of evidence and the balance of benefits and downsides (harms, burden and costs). Frequently these judgements are made implicitly rather than explicitly and judgements about the quality of evidence are confused with judgements about the balance of benefits and downsides. Many systems that are used to grade the quality of evidence and the strength of recommendations also confuse these judgements by equating the strength of recommendation with the quality of evidence, for example by grading recommendations for which there is high quality evidence as strong, without explicitly considering the balance of benefits and downsides.

Knowing the quality of evidence is essential, but not sufficient for making judgements about the strength of a recommendation. For instance, high quality evidence from well executed randomized controlled trials showed that oral anticoagulation administered for more than one year reduces the risk for recurrent thromboembolic events in patients after a first episode of spontaneous deep venous thrombosis. However, because oral anticoagulation is associated with harms (bleeding risk), burden (taking medication and monitoring anticoagulation levels) and cost (anticoagulation clinics or monitoring devices) the recommendation to anticoagulate all patients is weak because the benefits and downsides are finely balanced and individual patients will make different choices [2]. Both judgements about the quality of evidence and about the strength of a recommendation are complex and require consideration of a number of factors.

In this paper we addressed the following questions:

• Should WHO grade the quality of evidence and the strength of recommendations?

• What criteria should be used to grade evidence and recommendations?

• Should WHO use the same grading system for all of its recommendations?

Questions related to what evidence should be included, how it should be synthesized and reported are addressed in other papers in this series [3-5].

## What WHO is doing now?

WHO groups (e.g. WHO Europe) have acknowledged the need for evaluating or developing a grading system [6,7], and the Guidelines for WHO Guidelines recommend using a specific, uniform grading system [8]. However, this system, the Grading of Recommendations Assessment, Development and Evaluation (GRADE) approach, has scarcely been used within WHO [9,10]. Some WHO groups have developed their own grading systems [11,12], despite of the guidelines for WHO guidelines suggestion to use GRADE. Most have not explicitly graded either the quality of evidence or the strength of recommendations [13,14].

## What other organisations are doing

Most, but not all organizations that develop guidelines use a grading system to express the strength of a recommendation or the quality of evidence. For example, the US Preventive Services Task Force (USPSTF) uses a grading system that assigns one of three grades of evidence: good, fair, or poor [15]. The Task Force uses its assessment of the evidence and magnitude of net benefit to make a recommendation, coded as a letter: from A (strongly recommended) to D (recommend against). The UK National Institute for Health and Clinical Excellence (NICE) has not yet made a decision as to which grading system to use [16]. The Scottish Intercollegiate Guideline Network (SIGN) has developed its own grading system for application to SIGN guidelines [17]. The Australian Medical Research Council is currently developing a grading system that will probably include grading recommendations according to strength of recommendations and quality of evidence [18]. The US Task Force on Community Preventive Services uses a system in which the quality of the evidence of effectiveness links directly the strength of the recommendation [19,20]. Professional organizations use a variety of systems, many of them, however, based on two prominent grading approaches: the system derived from the Canadian Task Force on the Periodic Health Examination [1,21] and a successor of that system, the Oxford Centre for Evidence Based Medicine approach [22].

More recently, medical societies have begun to form collaborations within specialties to develop grading systems on their own. For example a group of specialty societies in rehabilitation sciences formed a panel to develop an approach to grading the quality of evidence and strength of recommendations [23]. This panel developed a set of criteria for grading the strength of both the evidence and the recommendation. Similarly, the world leading urology associations have come together to adopt a uniform grading system and approach that would be useful for urologists around the world rather than each association using a different grading system [24]. This latter collaboration named, Evidence Based Urology, is exploring using the GRADE approach. The GRADE approach is being used increasingly by organisations around the world [25-28], although in some cases with slight modifications [29]. It has been used for public health questions such as the pharmacological management of human influenza A(H5N1) infection (avian flu) [30], although it more commonly has been used for clinical questions up to now. A group of family practice and primary care journals has also developed a system to grade the strength of a recommendation [31].

## Methods

The methods used to prepare this review are described in the introduction to this series [32]. Briefly, the key questions addressed in this paper were vetted amongst the authors and the ACHR Subcommittee on the Use of Research Evidence (SURE). We did not conduct a full systematic review. We reviewed existing guidelines for guidelines to identify grading system currently in use. We also searched PubMed using (grading system) and (methods) (MESH headings/keywords) for systematic reviews and studies of methods for grading the quality of evidence. In addition, we searched databases maintained by the Agency for Healthcare Research and Quality (AHRQ, [33]) and the Guidelines International Network (GIN, [34]). These searches were supplemented with information obtained directly from guideline development organizations and our own files. Because of our involvement with organizations that produce guidelines and prior work with grading systems, in particular the GRADE system, we had in depth knowledge about several systems [25,28,29,35,36].

## Findings

We identified one systematic review dealing with the evaluation of grading systems. In 2002, the US Agency for Healthcare Research and Quality (AHRQ) published a systematic review of existing systems to grade the quality of evidence and strength of recommendations [37]. The AHRQ review considered 40 systems until the year 2000 that addressed grading the strength of a body of evidence. The important domains and elements for the systems to grade the strength of evidence that the authors agreed on were quality (the aggregate of quality ratings for individual studies, predicated on the extent to which bias was minimized), quantity (magnitude of effect, numbers of studies, and sample size or power) and consistency (for any given topic, the extent to which similar findings are reported using similar and different study designs).

More recently, independent work by the Canadian Optimal Medication Prescribing and Utilization Service (COMPUS) used a detailed process to evaluate and select an evidence grading system and expanded the work by AHRQ (while accepting it) until the year 2005 [38]. COMPUS, which identifies, evaluates, promotes and facilitates best practices in drug prescribing and use among health care providers and consumers in Canada [39], is a nationally coordinated program, funded by Health Canada and delivered by the Canadian Coordinating Office for Health Technology Assessment (CCOHTA, [39]). They assembled a working group of internal researchers, information specialists, methodology experts, and external researchers to update the work of AHRQ. COMPUS searched for and selected review articles for the period 2000 to 2005. This resulted in more than 3,000 citations for selection. Eleven review articles were selected for further analysis based on a priori selection criteria specified by the working group. Nearly 50 evidence grading systems were identified from the 11 review articles. Canadian and international experts in evidence evaluation methodology helped identify an additional 10 instruments or systems not included in the list of identified grading systems. The identified instruments and systems were evaluated using the AHRQ evaluation grids. The highest scoring instruments were the GRADE and the SIGN approach [38]. A second round of expert consultation and stakeholder input from all interested parties confirmed the selection of these instruments.

The GRADE system was developed through an intensive international collaboration of methodologists, guideline developers and clinicians and incorporates the factors identified in the AHRQ review and described above [35,36].

### Should WHO grade the quality of evidence and the strength of recommendations?

We did not identify published studies that compared graded with non-graded recommendations. The only evidence we are aware of are three unpublished studies. The first was conducted by UpToDate^®^, an electronic textbook, that asked a small group of users to compare graded with non-graded recommendations and explore – in a focus group setting – reasons for their answers (UpToDate^®^, personal communication). The second is our own study asking a small group of the general public interested in health care issues (Akl E, et al, manuscript in preparation). The third is a study by researchers in Norway who provided patients with back problems with graded evidence of the effects of alternative interventions graded with the GRADE approach. Users of the website intuitively understood the meaning of the quality grades for each outcome (Claire Glenton, personal communication). The findings of these evaluations suggested that users preferred graded over non-graded recommendations.

Despite the lack of stronger direct evidence, there is agreement among most guideline developers that grading the quality of evidence has advantages, because health care decisions involve a trade-off between likely benefits on the one hand, and downsides (harms, burden and costs) on the other hand [40]. To integrate these recommendations with their own judgment, guideline users need to understand the basis for the recommendations that guidelines offer them. A systematic approach to grading the strength of recommendations should minimize bias and aid interpretation about benefits and downsides. In addition, a systematic and explicit approach to making judgements about the quality of evidence and the strength of recommendations is likely to help prevent errors, facilitate critical appraisal of these judgements, and can help improve communication of this information [36].

### What criteria should be used to grade evidence and recommendations?

In a series of 16 international meetings and correspondence over five years the GRADE Working Group has derived a set of criteria to assess the quality of evidence (Table 1) and the strength of recommendations (Table 2) [25,29,35,36,41,42]. The GRADE system has several advantages over other systems including explicit definitions and sequential judgments during the grading process; a detailed description of the criteria for the quality of evidence for single outcomes and for the overall quality of the evidence; weighing the relative importance of outcomes; consideration of the balance between health benefits versus harms, burdens and cost; and the development of evidence profiles and summaries of findings. In addition the GRADE group is supported by an international collaboration [36]. The main limitation and criticism of the GRADE system is its complexity. Work in progress is addressing this limitation including the development of user friendly software to develop evidence profiles (G. Vist, personal communication and [26,29]).

**Table 1:** GRADE quality assessment criteria

**Quality of evidence**	**Study design**	Lower if *	Higher if *
High	Randomised trial	**Study quality**: -1 Serious limitations -2 Very serious limitations -1 Important **inconsistency** **Directness**: -1 Some uncertainty -2 Major uncertainty -1 **Sparse data** -1 High probability of **Reporting bias**	**Strong association**: +1 Strong, no plausible confounders, consistent and direct evidence** +2 Very strong, no major threats to validity and direct evidence*** +1 Evidence of a **Dose response **gradient +1 All **plausible confounders **would have reduced the effect
Moderate			
Low	Observational study		
Very low			

* 1 = move up or down one grade (for example from high to intermediate) 2 = move up or down two grades (for example from high to low)

** A statistically significant relative risk of >2 (< 0.5), based on consistent evidence from two or more observational studies, with no plausible confounders

*** A statistically significant relative risk of > 5 (< 0.2) based on direct evidence with no major threats to validity

**Table 2:** Decisions about the strength of a recommendation

**Factors that can weaken the strength of a recommendation**	**Explanation**
Lower quality evidence	Will create greater uncertainty about the size of the (relative) effects (benefits and harms)
Uncertainty about the balance of benefits versus harms and burdens	Uncertainty about the baseline risk, prevalence of a problem or health status, which could affect the size of the (absolute) effects
Uncertainty or differences in values	Uncertainty about the relative importance of the benefits and downsides to those affected, or differences in how important they are to different people, which could affect the balance between the benefits versus harms and burden
Marginal net benefits or downsides	The anticipated net benefits or downsides are small (and uncertain)
Uncertainty about whether the net benefits are worth the costs	Uncertainty related to lack of information about the cost or whether the resource expenditure is justified by the anticipated benefit

### Should WHO use the same grading system for all of its recommendations?

We did not identify evidence for or against using a single grading system for all types of recommendations, including clinical, public health and health policy recommendations. The arguments for and against using a single grading system are summarised in Table 3. The most important reasons for using a consistent system are a) minimising confusion amongst users of WHO recommendations; b) the risk of bias if groups can select a system that makes the quality of evidence and the strength of recommendations look better for their preferred interventions; and c) being intellectually honest about recognising the limits of the evidence rather than having a double standard. If an approach can be identified that is suitable across a wide range of interventions and contexts both methodologically and politically, the advantages outweigh the disadvantages.

**Table 3:** Pros and cons of using the same system for grading evidence and formulating recommendations for a wide range of health care interventions, including clinical and non-clinical interventions

Arguments for having a common approach	Arguments against having a common approach
• Having less demanding systems for some kinds of questions might result in false positive conclusions. • People with vested interests in particular interventions could choose the system that makes their intervention look best. • People with vested interests in particular evaluation approaches could choose the system that makes their preferred evaluation approach look best. • Having different systems for different types of interventions might be confusing. • It is intellectually honest to recognise the limits of evidence where this is appropriate. • Admitting the limitations of evidence, if this is appropriate, might promote more and better research.	• Having an infeasible system for some kinds of questions might result in false negative conclusions. • False negative conclusions due to inappropriate evaluation requirements may have negative political and health consequences; for example, effective programs that cannot be studied with randomised trials might experience funding cuts. • Interventions that cannot be studied with randomised trials might not be evaluated. • A single system may not discriminate adequately within the range of evidence that is appropriate to consider for clinical and non-clinical interventions. • A system that can adequately address evidence across a wide range of interventions and contexts may be overly complex.

Some developers and users of GRADE believe that GRADE can be consistently and usefully applied across clinical and non-clinical interventions, based on conceptual arguments and experience up to now applying this approach to a wide range of interventions, including public health and health system interventions. Others disagree because they believe it is unlikely to be an appropriate approach for some areas for the reasons summarised in table 3. There is not yet an empirical evidence base with which to mediate this disagreement for GRADE or any other grading system. Up to now GRADE has been used mostly for clinical interventions and few examples of its use with public health questions have been published. There is an ongoing international collaborative effort to apply the GRADE approach to public health and health systems interventions, and it is possible that modifications may be needed to ensure its usefulness for non-clinical interventions. For example, in one recent review of drug policies the authors felt that it was important to distinguish between different types of observational studies (interrupted time-series analyses and controlled before-after studies) when making judgements about the quality of evidence for important outcomes [43].

## Discussion

WHO has made a decision to use a grading system to grade the quality of evidence and strength of recommendations that is sensible and is being used widely, the GRADE system [36]. WHO has been involved in the development of this system from the beginning, and consideration has been given to the potential for application of the system to WHO guidelines in developing the GRADE approach. This might have been expected to facilitate the dissemination and adoption of this approach by WHO guideline developers. However, interest in GRADE workshops at WHO has been limited, there is not a tradition of grading the quality of evidence or strength of recommendations at WHO, and few resources have been invested in supporting the use of GRADE specifically, or in supporting more rigorous guidelines development methods generally.

More recently, however, the WHO rapid advice guideline panel for the pharmacological management of human infection with avian influenza A (H5N1) virus applied GRADE successfully [30] and several WHO guidelines are under development using GRADE (Sue Hill, personal communication). In general, the evidence that graded recommendations have advantages over non-graded recommendations is limited, but there are strong arguments, including the clear and transparent communication of how much confidence users can place in recommendations and the evidence underlying them. Another limitation is that both the quality of evidence and the strength of recommendations exist on a continuum. Categorization of quality into four categories and recommendations for or against treatments into two grades, strong and weak, may oversimplify complex health care recommendations, but guidelines consumers are generally likely to benefit from this simplification as they are most interested in which recommendations to follow.

## Further work

We have found a large body of work on the development and evaluation of various grading systems. Problems have arisen because many different grading systems exist. Future efforts should focus on forging a consensus on using a sensible and uniform approach to grade the quality of evidence and strength of recommendations, building on the work of the GRADE working group. Use of the GRADE system by WHO, as is currently recommended by the Guidelines for WHO Guidelines, could help by obtaining more experience, particularly with non-clinical interventions, contribute to improvements in the existing system, contribute to agreeing on a common international approach to grading of recommendations and help to ensure the quality and transparency of the judgements that are made across various groups that make recommendations on behalf of WHO. Development of software and a detailed manual to simplify the use of the GRADE system is underway and should facilitate the use of this system and its further development.

## Competing interests

ADO and AF work for the Norwegian Knowledge Centre forthe Health Services, an agency funded by the Norwegian government that produces systematic reviews and health technology assessments. All three authors are contributors to the Cochrane Collaboration. ADO and HJS are members of the GRADE Working Group. HJS is documents editor and chair of the documents development and implementation committee for the American Thoracic Society and senior editor of the American College of Chest Physicians' Antithrombotic and Thrombolytic Therapy Guidelines.

## Authors' contributions

HJS prepared the first draft of this review. AF and ADO authors contributed to drafting and revising it. All authors read and approved the final manuscript
